# Relaxin Affects Airway Remodeling Genes Expression through Various Signal Pathways Connected with Transcription Factors

**DOI:** 10.3390/ijms23158413

**Published:** 2022-07-29

**Authors:** Joanna Wieczfinska, Rafal Pawliczak

**Affiliations:** Department of Immunopathology, Medical Faculty, Medical University of Lodz, 90-752 Lodz, Poland; joanna.wieczfinska@umed.lodz.pl

**Keywords:** relaxin, RXFP1, airway remodeling fibrosis, asthma, rhinovirus

## Abstract

Fibrosis is one of the parameters of lung tissue remodeling in asthma. Relaxin has emerged as a natural suppressor of fibrosis, showing efficacy in the prevention of a multiple models of fibrosis. Therefore, the aim of this study was to analyze the aptitudes of relaxin, in the context of its immunomodulatory properties, in the development of airway remodeling. WI-38 and HFL1 fibroblasts, as well as epithelial cells (NHBE), were incubated with relaxin. Additionally, remodeling conditions were induced with two serotypes of rhinovirus (HRV). The expression of the genes contributing to airway remodeling were determined. Moreover, NF-κB, c-Myc, and STAT3 were knocked down to analyze the pathways involved in airway remodeling. Relaxin decreased the mRNA expression of collagen I and TGF-β and increased the expression of MMP-9 (*p* < 0.05). Relaxin also decreased HRV-induced expression of collagen I and α-SMA (*p* < 0.05). Moreover, all the analyzed transcription factors—NF-κB, c-Myc, and STAT3—have shown its influence on the pathways connected with relaxin action. Though relaxin requires further study, our results suggest that this natural compound offers great potential for inhibition of the development, or even reversing, of factors related to airway remodeling. The presented contribution of the investigated transcription factors in this process additionally increases its potential possibilities through a variety of its activity pathways.

## 1. Introduction

Airway remodeling is the most typical pathological feature of asthma, and it includes subepithelial fibrosis, with increased deposition of extracellular matrix proteins (ECMs), smooth muscle hyperplasia, goblet cell metaplasia, and neovascularization. These changes are involved in persistent airflow limitation and collectively lead to a thickened airway wall that exacerbates airway hyperresponsiveness (AHR), resulting in fixed airway obstruction. The mechanisms controlling the pathogenesis of airway remodeling remain poorly understood. Nevertheless, it is well-known that repeated episodes of airway inflammation may be associated with airway remodeling. Moreover, the remodeling process progresses independently of inflammation and occurs early in the airway disease development [1,2,3,4].

Airway infections caused by viruses (e.g., rhinovirus), which are also the trigger of exacerbations, may also have severe adverse outcomes in patients with established asthma [5,6,7].

The degree of fibrosis developing in tissues depends on the balance between production and degradation of the extracellular matrix. In the airway wall, collagen degradation is regulated by matrix metalloproteases (MMPs) and tissue MMP inhibitors (TIMPs); collagen production by myofibroblasts occurs in response to pro-fibrotic cytokines, such as TGF-β1 [4,8]. Kuo et al., demonstrated that rhinoviruses contribute to airway remodeling by promoting ECM deposition and induction of ECM gene expression [9]. Furthermore, data suggest that viral infection-induced cytopathic effects may reduce the cell proliferation rate in bronchial epithelium, which results in an impaired repair process. Skevaki et al., suggested that rhinoviruses contribute to the fibrotic component of airway remodeling. Moreover, fibroblast growth factor is induced by rhinoviruses in the airway epithelium [10].

Human Relaxin-2 is a dimeric peptide, pleiotropic hormone with recognized antifibrotic properties. It also plays key roles in both reproductive and non-reproductive processes [11,12]. Relaxin treatment has been demonstrated to inhibit fibrosis in a bleomycin-induced model of lung fibrosis by hindering TGFβ-induced matrix protein production and increasing the expression of the collagen-degrading enzymes and matrix metalloproteinases [13]. Furthermore, relaxin administration has been reported to prevent the development of collagen deposition associated with airway inflammation in ovalbumin (OVA)-sensitized guinea pigs and OVA-sensitized challenged BALB/c mice, highlighting the antifibrotic potential of exogenous relaxin therapy [13,14]. Relaxin has also been shown to reverse fibrosis in an OVA model and play tissue-specific roles in regulating ECM deposition in organs such as the kidney, heart, liver, and lung [15].

RXFP1 (relaxin/insulin-like family peptide receptor 1) is the only relaxin receptor known to be expressed in the lung, and its natural ligand is relaxin (RLN2) [16]. RXFP1 is expressed at very low levels and is not secreted from cells. In bronchial asthma, RXFP1 expression is reduced, as in idiopathic pulmonary fibrosis, suggesting its significant role in respiratory diseases, among others [17,18]. Apart from the anti-fibrotic properties, relaxin has been shown to present anti-inflammatory effects by reducing the influx of neutrophils and mast cells into injured organs or inhibiting histamine release by mast cells [19,20].

Though the exact role of NF-κB in airway remodeling remains unclear, this transcription factor has been implicated in the pathogenesis of asthma. Tully et al., demonstrated the importance of NF-κB activation within the bronchiolar epithelium in dust mite-induced inflammation and fibrotic airway remodeling [21]. Moreover, NF-κB activation has been suggested to be necessary for neutrophil elastase-induced secretion of TGFβ-1 from smooth muscle cells, and it is involved in epithelial-mesenchymal transition in the mouse model of asthma, showing an alleged role of NF-κB activation in smooth muscle cells in AHR and remodeling [22]. STAT3 has been demonstrated to be involved in even more processes connected to airway remodeling, such as smooth muscle cell proliferation, repair of epithelium, and fibroblast migration. Moreover, MAPKs and STAT3 signaling have been suggested to be integrated into a network, which would be pivotal for airway remodeling development [23]

The aim of this study was to analyze relaxin influence on remodeling-related gene expression in an airway remodeling model induced by rhinovirus (HRV-2 and HRV-16); we also analyzed the contribution of chosen transcriptions (NF-κB, c-myc, and STAT3) in relaxin action.

## 2. Results

**Figure 1:**
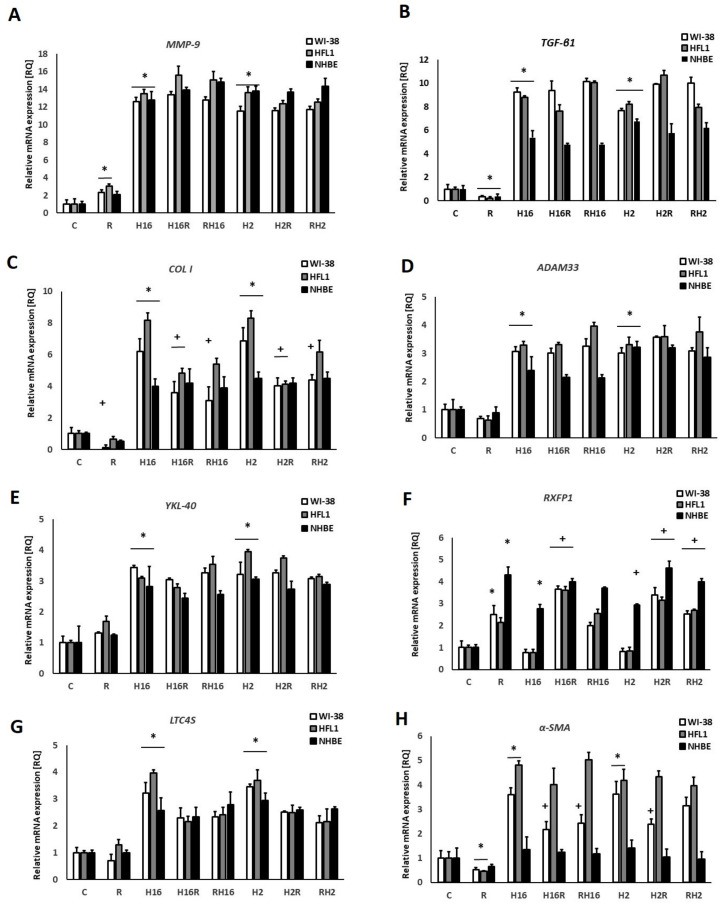
qPCR analysis showing the effect of relaxin (R) on airway remodeling-involved genes. Human Rhinovirus (HRV-2 or HRV-16) induced the expression of all genes analyzed, except for the RXFP1 (Relaxin Family Peptide Receptor 1) gene. Relaxin induced mRNA expression of MMP-9 (matrix metalloproteinase-9, (**A**)), but reduced the expression of TGF-β (Transforming growth factor, (**B**)) and collagen I (**C**). It also induced mRNA expression of RXFP1 (Relaxin Family Peptide Receptor 1, (**F**)); no effect of relaxin was observed in ADAM33 (ADAM Metallopeptidase Domain 33), YKL-40 (Chitinase-3-like protein 1), and LTC4S (leukotriene C4 synthase) mRNA expression (**D**,**E**,**G**). Finally, relaxin inhibited HRV-induced expressions of collagen I (**C**) and α-SMA (α-smooth muscle actin, (**H**). There were also no differences between the specimens when relaxin was added to HRV-infected cells or prior to the HRV infection: H16R, H2R—rhinovirus 16 and 2 added first, followed by relaxin—after 24 h, as well as RH16, RH2—relaxin added first, followed by rhinovirus-16 and 2—added after 24 h. * *p* < 0.05, in comparison to the control sample, and + *p* < 0.05 in comparison to the rhinovirus sample (HRV-2 or HRV-16, respectively); C is the control sample with medium only. *N* = 6, error bars—SEM.

Stimulation with relaxin caused a significant increase in the mRNA expression of the MMP-9 gene (234% and 306% for fibroblasts (WI-38 and HFL1) and 210% for epithelial cells, *p* < 0.05, Figure 1A).

Interestingly, relaxin significantly decreased the expression of TGF-β1 (91%), COL I (86%), and α-SMA (45%) mRNA (Figure 1B,C,H), and only in the case of TGF-β1, this effect appeared in all cell types tested (91% for WI-38, 87% for HFL1, and 80% for NHBE). In the case of collagen I, relaxin was effective only in WI-38 fibroblasts –47% decrease). For α-SMA, the decreasing effect of relaxin occurred in fibroblasts (WI-38–49% decrease and HFL1–55% decrease). There was no effect of relaxin on the expression of ADAM33, YKL-40, and LTC4S genes (*p* > 0.05, Figure 1D,E,G).

The infections with Rhinovirus-2 and -16 caused an increase in mRNA expression in all experiments. Interestingly, RXFP1 mRNA expression (Figure 1F) only increased, after rhinovirus infection, in epithelial cells (for 276%). In this set of experiments, we observed that relaxin also significantly increased the expression of the relaxin receptor RXFP1 mRNA (251% in fibroblasts and 430% in epithelial cells) in the presence of both serotypes of rhinoviruses (>200% *p* < 0.05), although in fibroblasts, the virus itself did not affect the expression of this gene. RXFP1 expression, after relaxin was added to HRV-infected cells (both serotypes used), was even greater than after relaxin alone (increase of 115% in WI-38 and 146% in HFL1). No significant differences were observed between the experiments. Relaxin was added to previously infected cells, and in the experiments, relaxin was added prior to infection.

**Figure 2:**
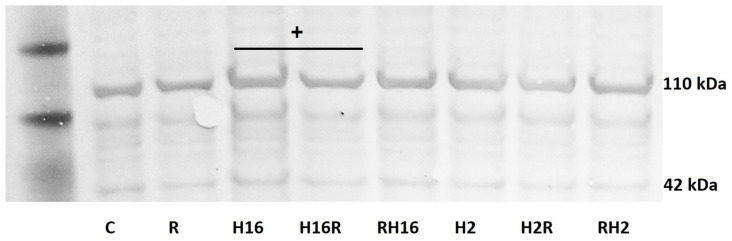
Immunoblot confirmation of relaxin effects. The results are from the HFL1 fibroblast cell line, presenting relaxin (R) influence on collagen I protein expression. Collagen type I (COL1) consists of two α1 (COL1A1) chains and one α2 chain (COL1A2, presented here, 110 kDa), which is a hallmark of fibrosis. The results mirror the expression obtained on the mRNA level. OD (Odds Ratio for H16 = 84 vs. OD for H16R = 72, + *p* < 0.05 in comparison to the rhinovirus sample (HRV-2 or HRV-16, respectively)); C—control sample with medium only. *N* = 3, β-actin (42 kDa) was an endogenous control. H16R, H2R—rhinovirus 16 and 2 added first, followed by relaxin—after 24 h, as well as RH16, RH2—relaxin added first, followed by rhinovirus 16 and 2—added after 24 h.

The study of protein expression has only shown statistical significance in the case of collagen I in fibroblasts (Figure 2). COLI expressions after the rhinoviruses were greater than the control sample (216% *p* < 0.05). Relaxin caused a significant reduction in the HRV-2-induced expression of collagen I but only when added to an existing infection (42% decrease, *p* < 0.05); Relaxin not only reversed, but also prevented, the upregulation of collagen 1. All other changes in protein expression were statistically insignificant (*p* > 0.05).

**Figure 3:**
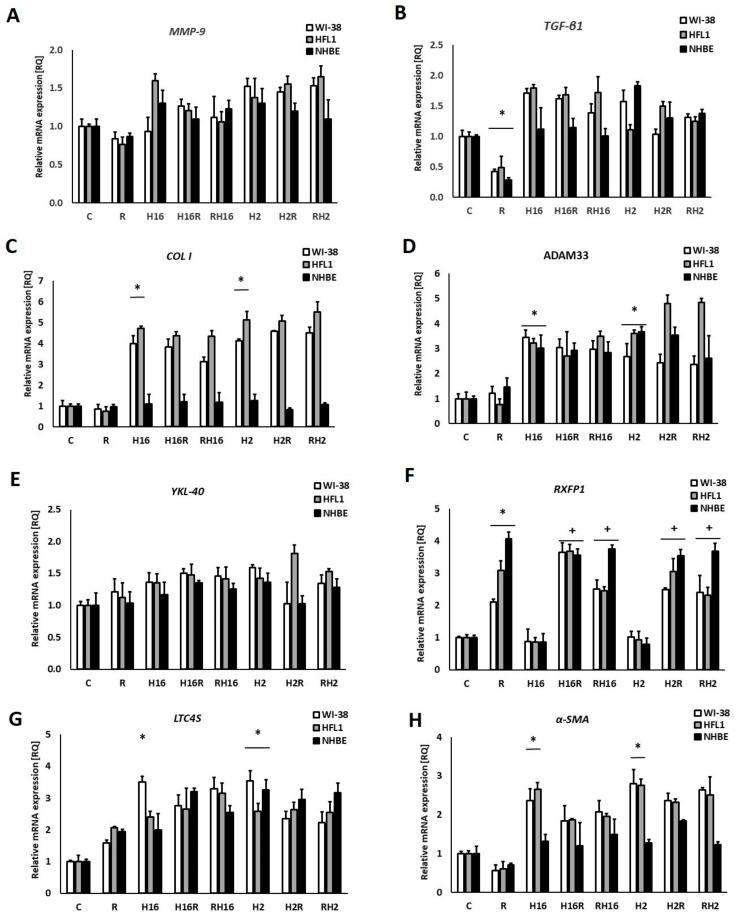
Effects of relaxin (R) in the NF-κB knockout conditions. Knockout of NF-κB (nuclear factor kappa-light-chain-enhancer of activated B cells) resulted in a lack of changes in MMP-9 (Matrix metalloproteinase 9) mRNA expression, either after relaxin or after HRV-2 (Human Rhinovirus) and HRV-16 treatment (**A**). Additionally, rhinovirus infections did not result in changes in the expression of MMP-9 (Matrix metalloproteinase 9, (**A**)), and TGF-β (Transforming growth factor beta, (**B**)) mRNA expression. Similarly, no significant effects of relaxin were observed in the mRNA expression of collagen I (**C**). No effects of relaxin have been observed in ADAM33 (**D**), and YKL-40 (Chitinase-3-like protein 1, (**E**)) mRNA expression, while relaxin increased mRNA expression of RXFP1 (**F**). In case of LTC4S (**G**) and α-SMA (α-smooth muscle actin, (**H**)), relaxin did not cause significant change of mRNA expression. No difference has been observed between the specimens when relaxin was added to HRV infected cells or prior to the HRV infection. H16R, H2R—rhinovirus 16 and 2 added first, followed by relaxin—after 24 h, as well as RH16, RH2—relaxin added first, followed by rhinovirus 16 and 2—added after 24 h. The data presented are normalized to the samples treated with control siRNA. * *p* < 0.05 in comparison to the control sample, and + *p* < 0.05 in comparison to the rhinovirus sample (HRV-2 or HRV-16, respectively); C is the control sample with medium only. *n* = 6, error bars—SEM.

siRNA silencing of transcription factors, when silencing the NF-κB transcription factor, showed no significant changes in mRNA expression of the MMP-9, and COL I genes were noted (Figure 3A,C), which suggests that this transcription factor has a significant impact on the expression of the above genes. Similar effects appeared in the study of α-SMA mRNA expression (Figure 3H). Nevertheless, the silencing of NF-κB only affected fibroblast cells. RQ values after relaxin, in epithelial cells, remained unchanged in comparison to the basal experiments (i.e., in comparison to Figure 1).

**Figure 4:**
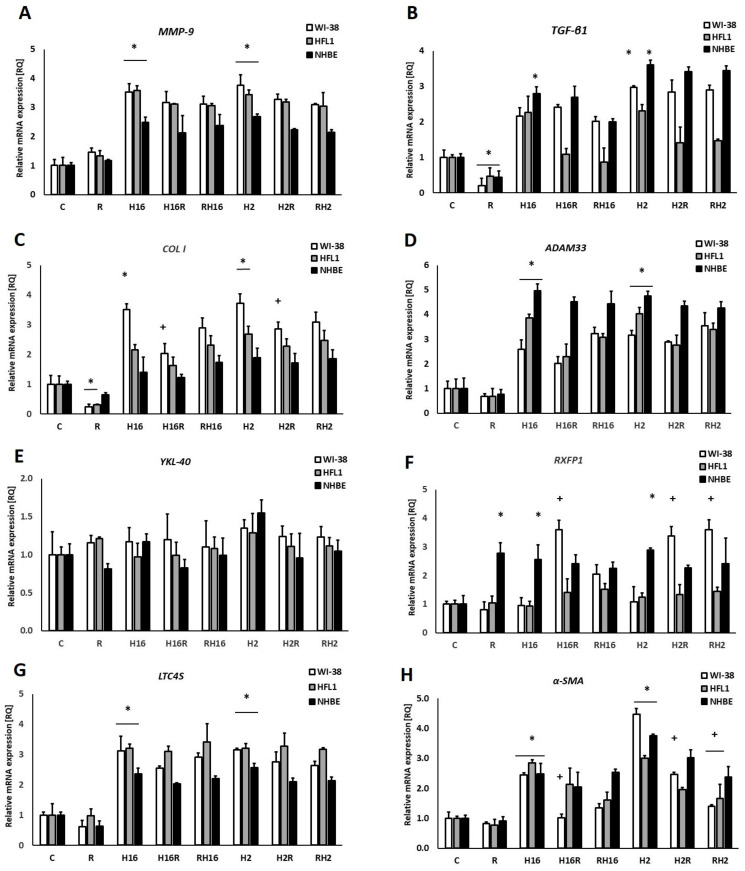
siRNA c-Myc knockout effect on Relaxin (R) in rhinovirus-infected cells. c-Myc transcription factor knockout inverted the relaxin (R) effect of a decrease in MMP-9 (Matrix metalloproteinase 9, (**A**)) expression. TGF-β (**B**), and collagen I (**C**) mRNA expression was significantly decreased by relaxin. Under the conditions used, no effect of relaxin or rhinoviruses were observed in aDAM33 (**D**), YKL-40(Chitinase-3-like protein 1) (**E**) and LTC4S (**G**) expression. Relaxin changed the expression of RXFP1 in epithelial cells (**F**), and in fibroblasts in α-SMA (**H**). No difference was noticed between the specimens when relaxin was added to HRV (Human Rhinovirus)-infected cells (H16R, H2R) or prior to the HRV infection. H16R, H2R—rhinovirus 16 and 2 added first, followed by relaxin—after 24 h, as well as RH16, RH2—relaxin added first, followed by rhinovirus 16 and 2—added after 24 h. The data presented are normalized to the samples treated with control siRNA. * *p* < 0.05 in comparison to the control sample, and + *p* < 0.05 in comparison to the rhinovirus sample (HRV-2 or HRV-16, respectively); C is the control sample with medium only. *N* = 6, error bars—SEM.

Silencing the transcription factor, c-Myc, caused an interesting change in the mRNA expression of the α-SMA gene (Figure 4H); despite the lack of a significant effect of relaxin itself, it decreased α-SMA expression induced by viruses (143% decrease for WI-38 and HRV-16; 309% decrease for WI-38 and HRV-2). Silencing of c-Myc also resulted in the lack of the previously observed effect: increasing MMP-9 mRNA expression in all tested cells (Figure 4A). Intriguing results were reported from experiments showing the mRNA expression of the RXFP1 gene: WI-38 fibroblasts (but not HFL1) showed increased expression of this gene under conditions of HRV infection (361% for HRV-16 and 338% for HRV-2, *p* < 0.05) with relaxin. Although alone, none of these factors induced a significant effect.

**Figure 5:**
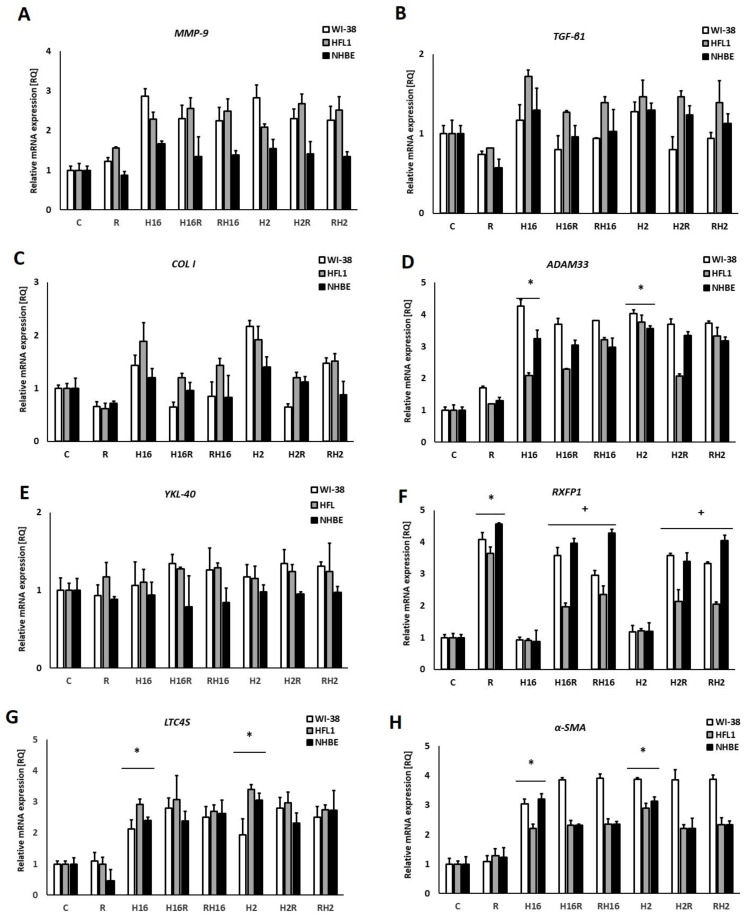
Real-time PCR results presenting the effect of relaxin on the genes contributing airway remodeling after STAT3 siRNA silencing. The knockdown of STAT3 reversed relaxin’s (R) ability to influence *MMP-9* (Matrix metalloproteinase 9, (**A**)), *TGF-β* (Transforming growth factor beta, (**B**)), *COL I* (collagen I, (**C**)), and α-*SMA* (α-smooth muscle actin, (**H**)) mRNA expression. The knockout did not change levels of *ADAM33* (ADAM Metallopeptidase Domain 33, (**D**)), YKL-40 (**E**) and *LTC4S* (leukotriene C4 synthase, (**G**)) mRNA expression in comparison to cells expressing *STAT3*. Relaxin increased mRNA expression of RXFP1 (**F**). Moreover, no difference were observed between the specimens when relaxin was added to cells infected with rhinovirus-16 (H16) and rhinovirus-2 (H2) or prior to the HRV infection. H16R, H2R—rhinovirus 16 and 2 added first, followed by relaxin—after 24 h, as well as RH16, RH2—relaxin added first, followed by rhinovirus 16 and 2—added after 24 h. The data presented are normalized to the samples treated with control siRNA. * *p* < 0.05 in comparison to the control sample, and + *p* < 0.05 in comparison to the rhinovirus sample (HRV-2 or HRV-16, respectively); C is the control sample with medium only. *N* = 6, error bars—SEM.

siRNA silencing of STAT3 transcription factor abolished all changes previously observed in TGF-β1 mRNA expression (Figure 5B). With both relaxin and two rhinovirus serotypes stimulation, no significant change in gene expression was noted (without silencing, these changes were significant). A similar effect was observed for the expression of collagen I, while the expression of MMP-9 mRNA after relaxin was also not changed, despite its clear stimulating effect in experiments without gene silencing (Figure 5A). STAT3 siRNA silencing did not cause any changes in RXFP mRNA expression in comparison to the basal experiments (Figure 5F, *p* > 0.05).

Protein expression analysis showed no statistically significant changes in this set of experiments (*p* < 0.05)

## 3. Discussion

It has been postulated that the development of subepithelial fibrosis in human asthma can drive remodeling processes, including airway epithelial thickening [24]. Mediators that regulate fibrosis in bronchial asthma are disrupted, which results in imbalanced production and degradation of ECM [25].

In this study, infections with two serotypes of rhinoviruses were used—HRV-16 and HRV-2—and resulted in increased mRNA expression of the genes analyzed (Figure 1A–H). These results stay in agreement with our previous study [26,27,28] and the literature, as it has been reported that infections caused by rhinoviruses are associated with an increase in airway remodeling mediators, e.g., TGF-α, TIMP-2, HGF, MMP-10, LIGHT, IL-1β, GF-1, PDGF, endothelin, and TGF-β [3,10,29]. Our aim was to analyze the effect of relaxin on the expression of airway-remodeling genes, depending on whether it was added before or after rhinovirus infection.

The mechanisms by which relaxin mediates its antifibrotic effects have not been fully identified. The most important physiologic function of relaxin appears to be the remodeling of ECM in both reproductive and non-reproductive tissues. In our study, relaxin induced MMP-9 mRNA expression. Nevertheless, relaxin did not influence MMP-9 mRNA expression in the conditions of rhinovirus infection. MMP-9 leads to the degradation of collagen, which induces infiltration of inflammatory cells through the basement membrane and ECM. These processes, along with accelerating collagen deposition, induce airway remodeling [30,31]. These processes induce airway remodeling [30,31]. Notably, silencing two of the analyzed transcription factors—NF-κB and STAT3—changed the mRNA expression levels of MMP-9 after rhinovirus infection (Figure 1A, Figure 2 and Figure 4A). Rhinovirus acts, at least in part, via NF-κB. Moreover, NF-κB-DNA binding activity was rapidly induced in RV-infected cells [32]. The NF-κB family of transcription factors regulates several of the tissue-remodeling genes, including MMP-9 [33,34]. It has been previously suggested that relaxin-induced tissue remodeling, with increasing MMP-9 expression, depends on NF-κB activation [33]. A recently published analysis of upstream antiviral response regulation revealed that the highest-ranking candidate transcription factors driving anti-rhinoviral response were NF-κB, STAT1, STAT3, and interferon regulatory factor 7 [35]. Furthermore, Xuan et al., have proved that activation of STAT3 adjusts and controls MMP-9 expression. Additionally, MMP-9 production induced by ceramide occurs via activation of the JAK2/STAT3 pathway [36,37]. MMP-9 is an important factor in airway remodeling and is closely related to an equally important cytokine in this phenomenon: TGF-β. TGF-β and growth factors also serve as inflammatory cytokines in the MMP-9 secretion and production. Moreover, knockdown of TGF-β1 expression from cells affects MMP-9 gene expression [2,38,39], and MMP-9 may induce TGF-β1 production in the airway epithelium through the cleavage of EGF and EGF-like ligands, as well as activating EGFR [40]. The above evidence suggests the existence of a feedback loop. TGF-β activates ERK-1/2 and p38 MAPK, which leads to the upregulation of matrix metalloproteinase MMP-9. There is lack of studies assessing the protein levels or activity of pro-fibrotic markers induced by rhinovirus. Nevertheless, Skevaki et al., demonstrated that rhinovirus infection induces bFGF release by airway epithelium, and it stimulates stroma cell proliferation, contributing to airway remodeling in asthma, and Mehta et al., identified three molecules that contribute to fibrosis and lung tissue remodeling—LIGHT, IL-1β, and TGF-β—which are induced by Rhinovirus [10,29].

In our study, relaxin decreased mRNA expression of TGF-β (Figure 1B), and these results remained unchanged despite NF-κB and c-Myc knockout (Figure 3B and Figure 4B). On the contrary, significance of the relaxin effect had been abolished when STAT3 was silenced with siRNA, which indicates the importance of this transcription factor in the pathway responsible for TGF-β expression (Figure 5B).

The majority of data demonstrate that STAT3 is frequently activated in many fibrotic systems and that TGF-β1 signaling induces phosphorylation and the activation of kinases that are known to activate STAT3. This signaling further suggests that STAT3 progressively modulates fibrosis by different mechanisms [41,42,43]. Unemori et al., showed that relaxin decreased TGF-β expression in fibroblasts. TGF-β1 is associated with, but also stored in, ECM. Apart from attenuating TGF-β1 expression, relaxin may also indirectly stimulate the synthesis of ECM by the protease-mediated release of TGF-β1 [44].

Royce et al., previously showed that relaxin inhibits the effects of TGF-β on the proliferation and differentiation of ECM-producing myofibroblasts [4]. By inhibiting TGF-β expression, relaxin seems to demonstrate a modulatory potential in a remodeling process [4], which was confirmed by our study. We also proved that relaxin increased the mRNA expression of MMP-9 but decreased collagen I expression (Figure 1A,C), which corresponds to the research results, showing that relaxin promotes MMP-induced collagen degradation as part of its collagen remodeling effects [45,46]. Relaxin decreased the secretion of collagen by untreated fibroblasts. However, it was also able to control collagen synthesis markedly in the conditions of significant collagen overexpression [47]. Importantly, in our study, relaxin also had a significant down-regulatory effect on the secretion of collagen. The effect of relaxin on the expression of rhinovirus-induced mRNA collagen was significant only in fibroblasts, not in epithelial cells (Figure 1C). This phenomenon might be connected with the fact that the subepithelial layer, which contains airway fibroblasts, might be activated by profibrotic cytokines, including the TGF-β1, for phenotypic shifts into highly contractile myofibroblasts (FMT-fibroblast-to-myofibroblast transition) [48]. Nevertheless, this is an important result, confirmed by a Western blot analysis (Figure 2), which shows the impact of relaxin on the significant factor of airway remodeling process. These results stay in agreement with the literature, as relaxin has been shown to decrease the production of pathological collagen through inhibition of its synthesis and secretion from myofibroblasts in different organs [44,49,50]. Moreover, data published, as well as the results of this work, suggest that relaxin is able to promote the expression of matrix metalloproteinase to enable the degradation of collagen accumulation [44,47,51,52,53]. These properties confirm the useful antifibrotic action of relaxin.

An important issue in this study is the different responses to relaxin of different cell types. Each of them specifically coordinates the control of the airway microenvironment during lung pathophysiological processes. Anatomical and functional interactions between fibroblasts and airway epithelial cells are known as the epithelial-mesenchymal trophic unit (EMTU), which is important in airway remodeling. Our study suggest that fibroblasts react stronger to the relaxin treatment than epithelial cells. These findings may suggest that the subepithelial fibrosis may contribute to remodeling changes to a greater extent compared to epithelium, thereby supporting the idea that fibroblast-derived collagen deposition may subsequently promote epithelial remodeling changes that exacerbate airway hyperreactivity.

**Figure 6:**
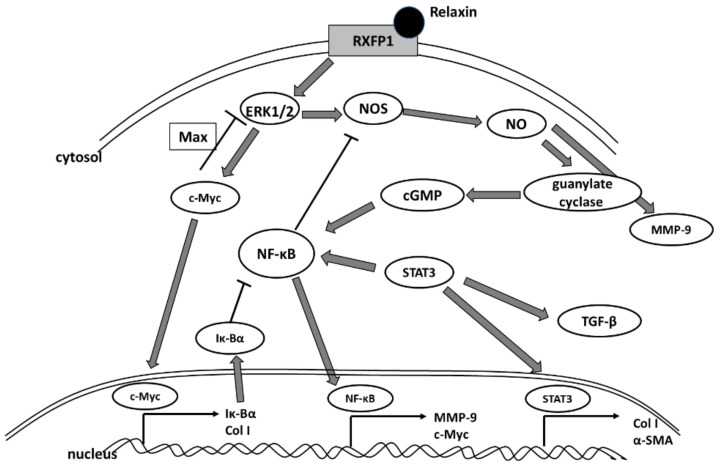
A schematic illustration of the proposed relaxin signaling at the cellular level.

In the present study, we demonstrate that the knockdown of NF-κB and STAT3 (but not c-Myc) abolished the effects caused by relaxin. Although it must be kept in mind that NF-κB and STAT3 pathways are not exclusively connected with either airway remodeling or the fibrosis process, and their actions have to be analyzed in a broader context. According to the literature, STAT3 is required for the increased COL1A2 expression observed in fibroblasts [54,55]. This transcription factor also operates at the post-transcriptional as well as the transcriptional level. Furthermore, Han et al., demonstrated that rhinovirus-induced inflammasome activation occurs in vivo, and that activation of inflammasome plays a pivotal role in rhinovirus-induced airway inflammation and hyperresponsiveness [56]. While activation of NLRP3 is crucial for host defense, its activation has also been associated with many other chronic diseases, including fibrosis [57]. The inhibitory effect of NF-κb silencing on relaxin activity may also be related to the fact that this protein is responsible for the transcription of the NF-κb inhibitor IκBα [58] (Figure 6). Inhibition of NF-KB, in turn, suppresses the action of NOS, which is necessary for the relaxin signaling pathway, and thus, its action becomes limited or inhibited.

NF-κB mediates induced collagen I expression and NALP3 inflammasome, which contributes to the development of bleomycin-induced pulmonary fibrosis and is also involved in this pathway [59]. On the other hand, relaxin was proven to inhibit NLRP3 inflammasome activity, which, apart from increasing interleukin-1β activity, plays a role in inhibiting NF-κB signaling: a crucial transcription factor that regulates many inflammatory genes. Therefore, relaxin action may be considered indirect in some circumstances [47,60,61].

Surprisingly, no effect of relaxin has been found in mRNA expression of ADAM33, YKL-40, and LTC4S (Figure 1D,E,G and Figure 4D,E,G). ADAM33 has been identified as an asthma susceptibility gene [62,63]. YKL-40 has also been suggested to contribute to asthma and tissue remodeling [64,65,66]. LTC4 synthase was used, in the study, as an inflammation marker. These data may be connected with the fact that the relaxin treatment lasted 24 h, which might be too short for some genes to react to relaxin action. Nevertheless, no data regarding such a connection may be found in the literature. Therefore we are the first researchers to confirm a lack of relaxin influence on these two airway-remodeling connected genes.

The relaxin activity of net matrix remodeling, within target tissues, is possibly determined by the levels of its receptors and downstream signaling initiated by the activation of receptors through the binding with hormone ligands [67]. RXFP1 signaling involves multiple pathways—which depend on the cell type—in fibrotic diseases. The relaxin/RXFP1 axis is dysregulated [68,69]. Relaxin-2 has been shown to signal, via RXFP1, myofibroblasts by suppressing TGF-β1 signal transduction and activity, which occurred at the level of Smad2 phosphorylation. Subsequently, it resulted in a decrease in myofibroblast differentiation induced by TGF-β1 and, further, myofibroblast-mediated ECM/collagen synthesis [47,70,71]. Our study shows that the effects of relaxin were stronger in epithelial cells than in fibroblasts (Figure 1F). Relaxin alone not only increased the mRNA expression of RXFP1 but it also increased the expression even more when added to HRV-infected cells. Interestingly, rhinovirus alone enhanced the expression of RXFP1 only in epithelial cells. A recent study showed that, in endothelial cells, relaxin administration may increase its own receptor expression (RXFP1) through epigenetic regulation, in the form of histone modifications, by attenuating TGFβ-pSMAD2/3 signaling in endothelial cells [72]. Royce et al., stress that the main site of expression of RXFP1 is the bronchial epithelium. Epithelial cells are highly secretory, which means that they may influence the underlying fibroblasts and myofibroblasts via cytokines and growth factors [73,74]. The data presented in this paper show that knockdown of NF-κB and STAT3 diminished the changes in the RXFP1 values between epithelial cells and fibroblasts (Figure 3F and Figure 5F), while c-Myc knockdown did not influence those RQ values (Figure 4F), suggesting differences in expression regulation between different cell types.

The research of both in vitro and in vivo models indicates that airway fibrosis may lead to further changes of airway remodeling, including those affecting the epithelium [1,75,76,77,78]. In lung diseases characterized by fibrosis, RXFP1 gene and protein expression was found to be reduced, and in biopsy samples taken from asthma patients RXFP1 was found to be lower [17,79].

α-SMA, has long been recognized as a surrogate marker of activated fibroblasts [80]; indeed all myofibroblasts express α-SMA, which well-characterized protein used for the assessment of activated fibroblasts in lungs and other tissues [81,82,83]. Studies using fibroblast-populated collagen lattices indicate that α-SMA expression increases the contractile activity of fibroblasts [84]. Our study shows that relaxin significantly decreased the mRNA expression of the α-SMA gene in fibroblasts, it also decreased rhinovirus-induced mRNA expression of this gene (Figure 1H). This situation remained similar in the conditions of c-Myc knockdown (Figure 3H). c-Myc/Max heterodimer may inhibit ERK1/2, which is thought to play a role in relaxin pathway (Figure 6). Possibly there may be some additional unknown pathway involved. In kidneys, c-Myc promotes tubulointerstitial fibrosis by upregulating integrin αv (ITGAV). This leads to TGF-β activation (whcn induces α-SMA COL I production) and increased extracellular matrix production [85]. On the other hand, according to Rosch and colleagues’ study, which was conducted in hernia patients, significant increase in c-myc expression correlated to the decreased collagen I/III ratios [86] and, in the recent study of Shen et al., demonstrated that c-Myc siRNA inhibited the Ang II–induced accumulation of ECM renal proteins, including fibronectin, collagen I, and α-SMA [85]. Possibly, in airway cells, the mechanism may be similar; however, little data is available regarding this issue.

siRNA silencing of NF-κB and STAT3 eliminated the changes observed in basal experiments (Figure 3H and Figure 5H). These data may suggest that relaxin might have more molecular aptitudes but also that modulation of transcription factors may influence some elements of the airway re-modeling process.

Fibroblasts are dynamic cells, and it must be kept in mind that the interpretation of the findings, derived from different cell models, requires understanding of the diverse features of fibroblasts in each model. This may possibly explain the different relaxin results in the two fibroblast cell lines used. Recently published data suggest that α-SMA-overexpressing cells display reduced contraction, which may be connected with weakened proliferative activity [87]. These effects are consistent with the known physiological role of relaxin in matrix remodeling, showing the decrease in α-SMA expression and suggesting that relaxin can down-regulate the activity of these matrix-producing cells [80]. Recently published data suggest that HRV infection may contribute the remodeling process by the release of chemoattractants that can stimulate directed migration of airway smooth muscle cells (ASMCs) [88]. Our results show that the different serotypes used in the study have similar efficacy in inducing the expression of genes related to airway remodeling. This may be related to the fact that both serotypes used enter airway cells via ICAM-1, a member of the immunoglobulin superfamily. To date, no data have been published demonstrating differences between HRV serotypes in airway remodeling participation. However, it is possible that, despite the differences in the initial phase of virus action/entry, its subsequent effect on airway remodeling may be similar despite the different serotypes, though this issue requires deeper research.

The aim of the study was not to analyze the effect of relaxin in the context of its use for therapeutic purposes but only to evaluate its effects as a form of recognizing the area of its action and the possibility of modulating certain processes by means of relaxin. We are aware of the limitations of our study, which was conducted utilizing cell lines and with narrow time for relaxin. Moreover, the lack of confirmation of the HRV infection—and only partial confirmation of the results on the protein level—are also drawbacks of the work. Additionally, strong effects of relaxin application under basal conditions may suggest that application of relaxin can be associated with potent side effects, including destabilization of the airway structure. This issue requires further investigation, as studies show that the lower dose of relaxin (25 μg/kg/day) induces skin thickness and benefits in other lung parameters. Nevertheless, at the dose of 100 μg/kg/day, no benefits of relaxin were observed [89]. No data showing airway structure destabilization are now available. Therefore, the interpretation of our study should also be considered with caution.

Relaxin-2 assembles to RXFP1 and acts via ERK1/2 kinase and, subsequently, NOS/NO-dependent signaling, leading to the activation of guanylate cyclase and cGMP production [70]. ERK1/2 is known to stabilize c-Myc [90], which is responsible for the expression of collagen I or Iκβ–NF-κB inhibitor. On the other hand, c-Myc/Max heterodimer inhibits phosphorylated ERK1/2 [91]. Nitric oxide synthase might be inhibited by the knockout of NF- κB [92] but stimulated by STAT3, which, in turn, is an important factor for TGF-β [93,94]. STAT3 is a transcription factor for fibrotic genes such as collagen I and α-SMA [42,54]. cGMP is generated as a result of relaxin activity, and its signaling pathway may inhibit NF- κB activation in innate immunity [95]. cGMP is cyclic guanosine monophosphate, ERK1/2 is extracellular-signal regulated kinase 1/2, NOS is nitric oxide synthase, RXFP1 is relaxin family peptide receptor 1, and TGFβ is transforming growth factor-β.

## 4. Materials and Methods

### 4.1. Cell Cultures

WI-38 and HFL1—fibroblast cell lines—were purchased from Sigma-Aldrich (St. Louis, MO, USA). The cells were grown in EMEM medium (WI-38) and HAM’s12 medium (HFL1) with an addition of 10% fetal bovine serum, 2 mM of L-glutamine, 1% of non-essential amino acids, and standard Penicillin Streptomycin solution (Sigma-Aldrich, St. Louis, MO, USA). The epithelial cell line—NHBE—was purchased from Lonza (Lonza Walkersville Inc. Walkersville, MD, USA) and cultured in BEGM Bronchial Epithelial Cell Growth Medium BulletKit (Lonza Walkersville Inc. Walkersville, MD, USA). The experiments (*n* = 6) were performed after reaching 80–90% confluence (passage three to eight) by the cells. The viability of the cells was assessed using Presto Blue (BD Pharmingen, Franklin Lakes, NJ, USA) and measuring the absorbance at 570 nm.

### 4.2. Virus Preparation and Cell Infection

Human rhinovirus (HRV) 16 and HRV-2 were purchased from the European Collection of Authenticated Cell Cultures (ECACC, Salisbury, UK). Ohio HeLa cells were infected until cytopathic effects were observed (multiplicity of infection (MOI) of 1 for both HRV serotypes). Approximately 0.5 mL of HRV was inoculated into subconfluent H1-HeLa monolayer cells in a T-182 flask. Upon adsorption (1 h at room temperature with rocking), 50 mL of HRV infection medium was added (MEM supplemented with 2% FBS, 20 mM HEPES, 1×non-essential amino acids, 10 mM MgCl_2_), and the infection was allowed to proceed at 33 °C in a 95% CO_2_ awaiting the monolayer that appeared to be completely involved with the cytopathic effect (CPE) one to three days post-infection). Afterwards, cells and supernatants were harvested after three cycles of freezing/thawing in order to rupture all membranes, which were then clarified by centrifugation, aliquoted, and stored at −70 °C. Both serotypes were titrated by making a logarithmic dilution and inoculating Ohio HeLa cells in 96-well plates. The highest dilution at which a CPE was detected, in at least half of the wells, was defined as the end-point titre. Inactivaton of HRVs in specimens were performed by exposing them to a temperature of 58 °C for one hour (Bossios et al., Clin Exp Allergy 2008), which was subsequently confirmed by a lack of HRV replication. The target fibroblast and epithelial cells were infected by an addition of 50 μL vehicle (medium) or HRV-16/HRV-2 (MOI = 1). The cells were incubated for 24 h (33 °C, 5% CO_2_).

### 4.3. Experimental Procedure

The cultures were exposed to both serotypes of rhinovirus—HRV-2 (minor) and HRV-16 (major)—for 24 h (33 °C, 5% CO_2_). Before or after infection, the cells were incubated with relaxin (100 ng/mL) for 24 h (37 °C, 5% CO_2_). The controls were treated with the medium. All the experiments were performed three times in duplicate (passages 3 to 9). The time-point of the relaxin effect was chosen out of three (6, 12, and 24 h, data not shown), consistent with the literature [96,97,98,99,100]. The time point of 24 h was the one causing the strongest response, which was visible in the analyzed genes expressions (viability—Figure S2).

### 4.4. RNA Isolation and cDNA Synthesis

Total RNA was isolated from the cells by utilizing a Total RNA mini kit (A&A Biotechnology, Gdynia, Poland). The RNA was then purified and stored at −80 °C, and reverse transcription (1 μg of total RNA) was performed using a High Capacity cDNA kit (Applied Biosystems, Foster City, CA, USA). The procedures were performed according to the manufacturer’s protocols.

### 4.5. Gene Expression Analysis

The expression of collagen I, MMP-9, ADAM33, TGF-β1, YKL-40, LTC4S, RXFP1 and α-SMA were assessed with qPCR technique. TaqMan gene expression assays were used for the selected genes—collagen I—Hs00164004_m1, MMP-9—Hs00957562_m1, ADAM33—Hs00905552_m1, TGF-β1—Hs00998133_m1, YKL-40—Hs01072228_m1, LTC4S—Hs01073145_m1, RXFP1—Hs01073145_m1, α-SMA—Hs05005339_m1, and β-actin—Hs99999903_m1 (Life Technologies, Carlsbad, CA). Each sample was measured in triplicate, and the gene expression was used with the 2^−ΔΔCt^ method. The results were normalized to an endogenous reference gene (β-actin—Hs99999903_m1). LTC4 synthase was evaluated as an inflammation marker and α-SMA as a fibroblast-myofibroblasts transformation marker. By comparing RQ (relative quantification, 2^−ΔΔCt^), the fold change in mRNA expression was calculated.

### 4.6. Protein Isolation and Immunoblotting

The protein extraction was conducted with the RIPA protein extraction buffer (Sigma-Aldrich, St. Louis, MO, USA), supplemented with a protease inhibitor cocktail (Sigma-Aldrich, St. Louis, MO, USA), and determined by the BCA Protein Assay Kit (Pierce Thermo Scientific, USA). The electrophoresis was performed utilizing 10 µg of protein in denaturing polyacrylamide 4–20% NuPage gel (Invitrogen, Carlsbad, CA, USA) for 60 min (140 V and 110 mA). Then, the specimens were transferred into a nitrocellulose membrane with the eBlot Protein Transfer System (Genscript, Piscataway, NJ, USA). The membrane was incubated for one hour at room temperature, with 5% nonfat milk dissolved in TBST and incubated with primary mouse antibodies for 12 h at 4 °C (TGF-βa sc-130348, 1:1000, MMP-9 sc-393589 1:1000, COL I sc-393573, 1:500, ADAM33 sc-514055 1:500, YKL-40 sc-393590 1:500, α-SMA sc-53142 1:2000, ACTB sc-47778 1:1000 (Santa Cruz Biotechnology, Dallas, CA, USA), LTC4 MOB-2181z 1:500 (Creative Biolabs, New York, NY, USA), RXFP1 MAB8898 1:1000 (Biotechne, Minneapolis MN, USA) and then, with goat secondary anti-mouse IgG antibodies, conjugated with alkaline phosphatase for 90 min at room temperature. The antibodies were purchased from Santa Cruz Biotechnology, Dallas, USA. The bands on the membrane were developed using a BCIP/NBT alkaline phosphatase substrate (Merck Millipore, Darmstadt, Germany), and after that, they were analyzed with Image J 1.49 software (Wayne Rasband, National Institutes of Health, Bethesda, Washington, MD, USA).

### 4.7. siRNA Silencing of Transcription Factors

Silencer siRNA Transfection Kit (Thermo Fisher Scientific, Waltham, MA, USA) was used for the knockdown of selected genes (NF-κB, c-Myc and STAT3), according to the manufacturer’s instructions (Figure S1). The transcription factors were selected according to the literature, as the factors involved in the process of airway remodeling, but also as suggested elements of the relaxin pathway in this process [23,101,102,103,104,105]. The cells were treated with 20 nM siRNA mixture against NF-κB (NCBI accession no. NM_001145138.1), c-Myc (NCBI accession no. NM_002467.4) and STAT3 mRNA (NCBI accession no. NM_003150.3), (Thermo Fisher Scientific, Waltham, MA USA) for 48 h. The same concentration of scrambled siRNAs was used as the negative control. The knockdown efficiency was evaluated after 48 h of transfection. The measurement of gene knockdown was analyzed according to the manufacturer’s protocol by qPCR. The data presented are normalized to the samples treated with control siRNA.

### 4.8. Statistical Analyses

The results were analyzed with Statistica software (StatSoft, Tulsa, OK, USA). The Shapiro-Wilk test and Levene’s test were, respectively, used to check the distribution of data, as well as the equality of variances. Significant changes were calculated using the one-way ANOVA test with TukeyHSD (Tukey’s Honestly-Significant Difference) post-hoc test. *p* values < 0.05 were considered to be statistically significant. Data are presented as RQ (for qPCR experiments) or OD (odds ratio, for immunoblot experiments); Figure 3 and Figure 4 show that comparisons are made within groups, to control sample or to rhinovirus (2 or 16) sample. Details are included in the figure legends.

## 5. Conclusions

Airway remodeling is a very complex process, and it is impossible to require relaxin to stop the process, but this study was an attempt to point out some directions that may be useful for further research.

In conclusion, our results confirm and extend the knowledge regarding relaxin as a promising therapeutic agent in fibrotic diseases. As shown in this study, relaxin might protect, and even reverse, some changes that lead to airway remodeling. Moreover, the analysis of the influence of selected transcription factors on the effect of relaxin indicates new pathways that may be useful in the process of considering the potential therapeutic aptitudes of relaxin.

## Data Availability

The data presented in this study are available on request from the corresponding author. The data are not publicly available due to privacy reasons.

## Supplementary Materials

The following supporting information can be downloaded at: https://www.mdpi.com/article/10.3390/ijms23158413/s1.
