# Supplementary material for: Relaxin Affects Airway Remodeling Genes Expression through Various Signal Pathways Connected with Transcription Factors
Source: Int J Mol Sci. 2022 Jul 29;23(15):8413. doi: 10.3390/ijms23158413 (PMC9368845; doi:10.3390/ijms23158413)
Supplement: Supplementary file 1 [file ijms-23-08413-s001.zip › ijms-1777051-supplementary.pdf]

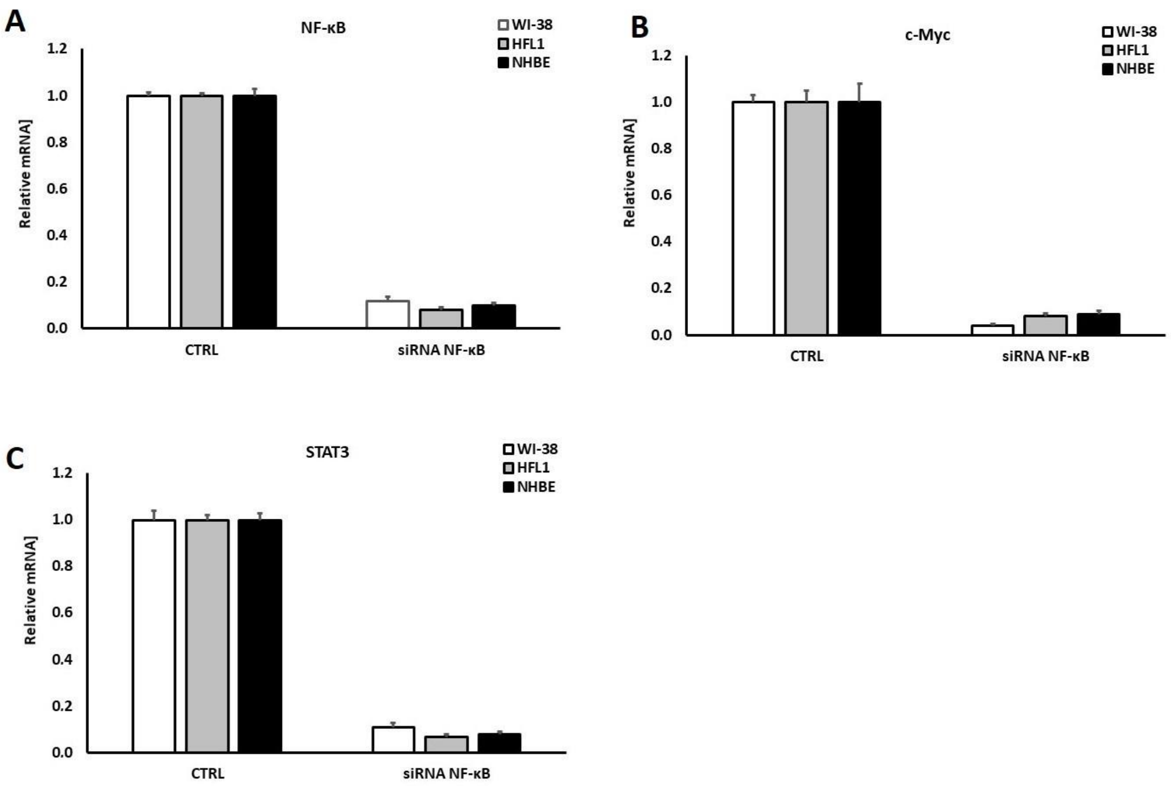

Figure S1. Knockdown efficiency of siRNA experiments

The transfections were performed when the cells were ~70% confluent. Relative mRNA expression, measured by RT-qPCR was determined, universal negative siRNA was utilized. siRNA transfection of NF-κB (A), c-Myc (B), STAT3 (C) in fibroblasts (WI-38 and HFL1) and epithelial cells (NHBE). Data represent mean of means +SEM (n=3).

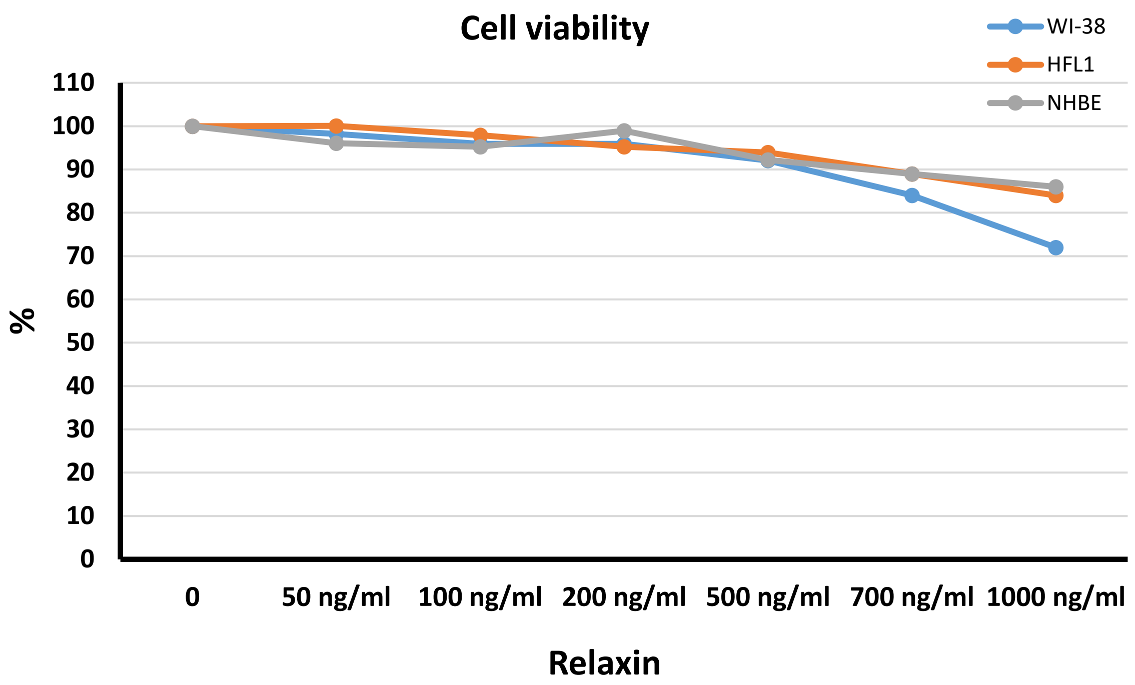

Figure S2. Cell viability after Relaxin-2 incubation.
